# Occupational Skin Diseases among Building Construction Workers in Dar es Salaam, Tanzania

**DOI:** 10.5334/aogh.3102

**Published:** 2021-09-23

**Authors:** Rabia Yussuf Esmail, Gloria H. Sakwari

**Affiliations:** 1 Tutor-School of Hygiene, Ministry of Health Community Development, Gender, Elderly and Children, TZ; 2 Lecturer-Environmental and Occupational Health Dept, Muhimbili University of Health and Allied Sciences, TZ

## Abstract

**Background:**

Amongst established growing industries worldwide, the construction industry contributes about 7.5% of the world labor force and 16.4% of global occupational diseases and accidents. A variety of activities are practiced in construction work, such as masonry, painting, welding, carpentry, plastering, concrete and cement mixing. These may eventually lead to exposures that can subject the workers to risks of developing occupational skin diseases. Most studies done on the construction industry in Tanzania have focused on effects such as injuries, respiratory problems and ergonomics; very little research has been done on skin diseases.

**Objectives:**

The study aimed at assessing the prevalence of occupational skin diseases, associated factors and preventive measures among building construction workers in Dar es Salaam, Tanzania.

**Methods:**

Construction sites and participants were selected using simple random sampling. The Nordic Occupational Skin Questionnaire (NOSQ) was adapted and used for assessing the development of skin diseases among constructions workers. Analysis of categorical variables, associated factors and skin preventive measures was done using Chi-square tests. Bivariate and multivariate logistic regression analyses were performed to determine odds ratio and adjusted odds ratio for factors indicating an influence on the occurrence of skin diseases.

**Findings:**

The study consisted of 420 building construction workers from different sites with a mean age of 32.7 years. The participants were masons, assistant masons and carpenters. The mean work duration was 6 years. Occupational Skin diseases were prevalent in 228 (54%) workers. Carpenters had a higher prevalence of skin diseases 12(67%), followed by assistant masons 43 (64%). Timely provision of safety trainings and of PPE utility, training and guidance lowered the occurrence of skin diseases.

**Conclusion:**

A high number of construction workers experienced skin diseases, especially those who have worked for more than 4 years. Workers who received safety training before the work shift had lesser prevalence of skin diseases compared to those who did not. Receiving proper information on PPE usage and proper wearing of gloves had a protective effect.

## Introduction

The construction industry is crucial in any country’s economy. It creates employment for all levels in the society and contributes over 50% to gross national capital formation [1]. In recent decades, the construction industry has been amongst the fast-growing industries worldwide. The industry contributes to about 7.5% of the world labor force and 16.4% of global occupational diseases and accidents [2]. The industry has demanding tasks and is termed the most dangerous working environment due to its different kinds of activities and working environments [3]. Thus, construction workers are regarded as having extremely demanding jobs, and the industry itself is tagged as 3-Ds: dirty, dangerous and difficult [2].

The construction industry involves different kinds of activities, such as masonry, welding, painting, paving, plumbing, electrical fixing, roofing, plastering, carpentry and others, which combine to form a sector. In these activities, workers work with glues, cement powder, thinners and other solvents, which may lead to health effects, including dermatological disorders [4]. The condition has been documented by various studies, including a cross-sectional study in Ahmedabad and Vadodara that revealed out of 92 construction workers, 47.8% reported having skin-related symptoms, such as frictional callosities in palm; dry, fissured and scaly skin with lesion and ulcers on hands [5]. A higher prevalence of skin diseases (59.5%) has also been reported among construction workers in Dhaka, Bangladesh [6].

In Tanzania, the construction industry has grown to recruit many due to the demand of services needed by residents [7]. However, occupational health and safety in this industry is still a critical issue, like any other developing country. Despite the presence of regulatory bodies, such as the Occupational Safety and Health Authority (OSHA), the health and safety of the workers are jeopardized due to a poor assessment on the impact, poor working environment, and weak enforcement of legislation and regulation, as well as underreported situations in the industries [8]. In addition, challenges such as poor assessment tools and few registered construction companies that provide services are among factors that worsen the health and safety issues in the construction industry in Tanzania [9]. This led to a reduction in the information needed on the severity of certain health hazards and occupational diseases, including occupational skin diseases. Thus, this study aims to determine the prevalence of skin diseases, associated factors and preventive measures used by the workers.

## Methodology

### Study aim and design

This study aims to assess the prevalence of skin diseases, associated factors and preventive measures used by workers in the building construction industry. This was a cross-sectional study that took place in Dar es Salaam. Data was collected from April 2016 to July 2016.

### Study population

The participants were construction workers from registered firms who worked in brick laying, concrete and cement mixing, plastering, floor laying, terrazzo, tile setting. These were grouped in the job categories of masons and assistant masons; carpentry was categorized as carpenters.

### Sample size

Sample size was calculated using the following formula for a single proportion:

n = Z^2^P(1–P)/e^2^,

Where

n: minimum sample size

Z: confidence interval at 95%

P: proportion of construction workers with skin problems is assumed as 50% and margin of error is 5% at 95% confidence interval.

{\rm {n}} = \frac{{{{1.96}^2}*50\left({100 - 50} \right)}}{{{5^2}}}

n: 384

Adjusting for a 10% non-response rate, the sample size is 426, so a sample size of 426 building construction workers was taken for this study.

From this calculation, 420 construction workers responding from 3 different districts in Dar es Salaam were recruited.

### Sampling procedure

The construction sites were randomly selected from a list of construction projects obtained from the Contractors Registration Board of Tanzania (CRB). The list contained construction projects that were done in Tanzania. Building construction projects that were indicated to be in Dar es Salaam were randomly picked from the list, aiming to have at least 10 to 14 building construction projects from each district.

### Data collection

Study participants were selected randomly on the day of data collection. All those who were present at the site had an equal chance of participating in the study. The standardized Nordic Questionnaire for assessment of Skin disease (NQS) was adopted and modified to fit Tanzania settings [10]. The NQS, which was used in countries like Ethiopia and the United Kingdom, has been found to have high validity and reliability when administered face to face.

The questionnaire was pretested before data collection and errors were clarified accordingly. The questions were translated from English into Swahili, the local language used by the majority of Tanzanians.

Data was collected by the principal investigator along with two research assistants who had knowledge of occupational health. A medical doctor (a registrar) assisted in verifying self-reported information on skin disease by performing physical examinations of the workers, who answered ‘yes’ on the questionnaire.

The dependent variable for this study was occupational skin disease, and the independent variables were the factors presumed to be associated in causing the disease:

- **Sociodemographic factors**: Sex, age, religion, ethnicity, marital status, level of education, monthly salary, employment condition, work experience.
- **Personal factors**: Improper Use of PPE, on-the-job training, working years on the current section, awareness of health and safety issues, information on PPE (types and utility), behaviour (habit of proper cleanliness, such as hand washing).
- **Work environment determinants**: Health and safety information, health and safety training, workplace supervision, work section, PPE utilization.
- **Managerial/institutional factors**: Poor provision of PPE, soap and water availability, enforcement of regulations, work instructions.

Ethical clearance for conducting this study was sought from Muhimbili University of Health and Allied Sciences (MUHAS) Institution Review Board (IRB).

At the construction site, permission to conduct the research was approved by the Contractors Registration Board through the provision of a supportive introductory letter, which explained the purpose of the study and requested participation.

Participants were assured of confidentiality of all the information they provided, and they were provided with the option to terminate their participation at any time in the course of the study, regardless of the consent signed for participation.

Data analysis was done using Windows Statistical Package for Social Science (SPSS) version 20. Preliminary data was checked for missing entries and outliers. Descriptive statistics were done to determine the prevalence of skin disease and other characteristics. The degree of association between the dependent and independent variables were tested using the Pearson chi-squared test for categorical variables (Fisher’s exact test was used when any expected number was less than 5). A value of p < 0.05 at 95% confidence interval was considered statistically significant. For any variable that had more than two by two table, contingency coefficient was used to set significant value.

A bivariate logistic regression was conducted for every single determinant, followed by a multivariate logistic regression for different predictors of skin disease, while adjusting for age.

## Results

### Prevalence of skin diseases by sociodemographic characteristics

**Figure 1:**
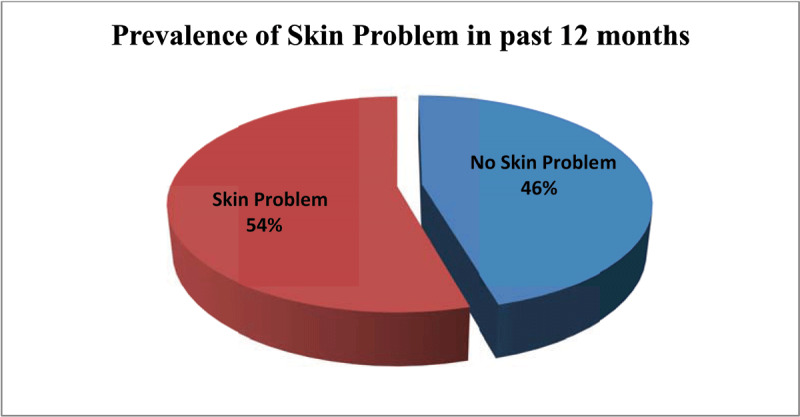
Prevalence of occupational skin disease among building construction workers, in the past 12 months.

The prevalence occupational skin diseases among the construction workers was 54% (***Figure 1***). Construction workers in the age group of 36 and above had a high prevalence of occupational skin diseases (83; 63.4%), followed by the age group of 31–35 (55.1%), compared to age groups of 26–30 and 25 and below, which had a low prevalence of (72; 52.2%) and (30; 41.1%), respectively (***Table 1***).

**Table 1:** Prevalence of skin diseases by sociodemographic characteristics and preventive practices (n = 420).


CHARACTER	SKIN DISEASE		TOTAL	P-VALUE

	PRESENT (N, %)	ABSENT (N, %)		

**Age group (years)**				

25 and below	30(41)	43(59)	73	0.021

26 to 30	72(52)	66(48)	138	

31 to 35	43(55)	35(45)	78	

36 and above	83(63)	48(37)	131	

**Education level**				

Non formal	13(62)	8(38)	21	0.096

Primary school	176(57)	135(43)	311	

Secondary school	39(44)	49(56)	88	

**Job category**				

Mason	169(55)	140(45)	309	<0.001^$^

Assistant Mason	43(64)	24(36)	67	

Carpenter	12(67)	6(33)	18	

Others^1^	4(15)	22(85)	26	

**Employment duration (years)**				

Below 2	58(45)	71(55)	129	<0.001^$^

2 to 3	60(49)	63(51)	123	

4 and above	110(66)	58(35)	168	

**Preventive Practices**				

**Receive safety training on work**				

Yes	100(47)	114(53)		0.002

No	128(62)	78(38)		

**Period of safety training**				

Before start of work	57(42)	78(58)		

During start of work	43(54)	36(46)		0.001^$^

None	128(62)	78(38)		

**Safety inspection done by site managers**				

Yes	126(51)	120(49)		0.134

No	102(59)	72(41)		

**Provision of personal protective equipment**				

Yes	175(52)	160(48)		0.095

No	53(62)	32(38)		

**Provided with hand washing facilities on site**				

Yes	208(54)	179(46)		0.448

No	20(61)	13(39)		

**Frequency of hand washing (frequency/day)**				

5	148(52)	135(48)		0.852

6–10	30(59)	21(41)		

11–20	17(61)	11(39)		

Do not wash hands	11(52)	10(48)		


1-Painters, plumbers: Chi-squared test, * Fishers exact test, $ Contingency coefficient.

A majority of those who reported having non-formal education experienced skin diseases more in comparison with primary and secondary education levels (61.9%, 56.6% and 44.3%), respectively, though there was no statistically significant association between experiencing skin problems and basic education level (p = 0.096) (***Table 1***).

Carpenters and assistant masons had a high prevalence of skin problems (12; 67% and 43; 64%, respectively), followed by masons (169; 55%). The job category of construction worker proved to be highly associated with an increase in skin diseases (p < 0.001). Exposure to the field in terms of work duration proved to be amongst the factors associated with the occurrence of skin diseases (p < 0.001). Construction workers with more than four years of experience demonstrated a high prevalence (110; 66%) compared to those who had worked for two to three years or less (60; 49% and 58; 45%, respectively) (***Table 1***). Prevalence of skin diseases did not differ between male (220; 54%) and female (8; 57%) workers; there were few female workers on site (***Table 1***).

### Prevalence of skin diseases by preventive measures

Construction workers who did not receive safety training had a high prevalence of skin problems (128; 62%) compared to those who got safety training before work began (p < 0.001). Those who received training during work had a higher prevalence (43; 54%) compared to those who received training before they started work (57; 42%), which was a significant factor for the occurrence of skin diseases (p = 0.001) (***Table 1***). Places where safety inspection was not done had a high prevalence of skin diseases (102; 59%) in comparison to places where site managers do not do inspection, although the difference was not statistically significant. Workers who did not receive training and guidance on PPE use had a higher prevalence (151; 59%) than those who had training (77; 46%). Construction workers who never wore gloves had a higher prevalence of skin problems (106; 60%) compared to those who use PPE always or at some point in time (p = 0.04) (***Table 1***).

### Reported skin problem-related symptoms and body area affected

Construction workers were affected with different symptoms and on different body parts in relation to their working area/job tasks. Almost all the skin-related symptoms showed in masons as a high prevalence of scales (40; 82%) followed by redness (21; 81%) and fissures (60; 66%). While assistant masons had a lower prevalence between 19% and 25%. In addition, the most affected body parts were the forearm (80; 78%) and hands (49; 69%) for masons, followed by assistant masons and carpenters who were affected on their hands (7; 10%) (***Table 2***).

**Table 2:** Distribution of respondents by skin-related symptoms and body area affected by job category (n = 228).


CHARACTER	JOB CATEGORY N (%)			

	MASON	ASSISTANT MASONS	CARPENTERS	OTHERS^1^

**Skin-related symptoms**				

Redness	21(81)	5(19)	0(0)	0(0)

Dry skin	48(71)	17(25)	3(4)	0(0)

Fissures	60(66)	23(25)	9(9)	0(0)

Scales	40(82)	5(10)	2(4)	2(4)

Blisters/Ulcers	40(73)	10(18)	5(9)	0(0)

Itching	56(76)	14(19)	4(5)	0(0)

Prickling	18(75)	6(25)	0(0)	0(0)

**Body area affected**				

Forearms	80(78)	17(17)	4(4)	1(1)

Hands	49(69)	26(21)	7(10)	0(0)

Upper limbs	43(73)	14(23)	1(2)	1(2)

Face	23(55)	16(38)	3(7)	0(0)

Lower limbs	46(84)	6(12)	1(2)	1(2)

Neck	23(51)	17(38)	4(9)	1(2)


^1^ Painters and plumbers.

### Unadjusted odds ratios (95% CI) for logistic regression between factors and occurrence of skin diseases among building construction workers

Education level seems to have an influence on the development of skin diseases, where those with a primary education level had significantly higher odds of developing skin diseases compared to those with secondary education (OR 1.63; 1.02–2.64). Construction workers with non-formal education had higher odds of developing skin diseases (OR 2.042; 0.77–5.42) compared to those with secondary education, though this was not significant. Workers who were carpenters had high odds of developing skin diseases (OR 11; 2.59–46.78), followed by assistant masons (OR 9.854; 3.04–31.96) and masons (OR 6.64; 2.24–19.72). Construction workers who worked for more than four years showed high odds of skin diseases (OR 2.32; 1.45–3.72) in comparison with those who worked for 3 years and below. Workers who did not receive safety training education had odds of developing skin diseases with odds of 2.25 (1.44–3.49) when compared with those who received training at some point (***Table 3***).

**Table 3:** Unadjusted and adjusted odds ratios (95% CI) for logistic regression between factors and occurrence of skin diseases among building construction workers.


VARIABLE	UNADJUSTED ODDS RATIO	95% CI(LOWER LIMIT–HIGHER LIMIT)	*ADJUSTEDODDS RATIO	95% CI(LOWER LIMIT–HIGHER LIMIT)

**Age group (years)**				

25 and below	1			

26 to 30	1.564	0.881–2.775		

31 to 35	1.760	0.924–3.358		

36 and above	2.478	1.379–4.454		

**Education level**				

Secondary school	1		1	

Non formal	2.042	0.769–5.418	1.202	0.425–3.399

Primary school	1.638	1.017–2.638	1.316	0.780–2.219

**Job category**				

Others^1^	1		1	

Mason	6.639	2.235–19.720	6.745	2.195–20.727

Assistant mason	9.854	3.038–31.960	9.666	2.843–32.868

Carpenters	11.000	2.587–46.779	11.000	2.588–54.616

**Employment duration (years)**				

Below 2	1		1	

2 to 3	1.166	0.710–1.913	1.121	0.650–1.934

4 and above	2.322	1.450–3.717	2.214	1.283–3.820

**Safety training on work**				

Yes	1		1	

No	1.871	1.268–2.761	1.452	0.922–2.285

**Period of safety training**				

Before start of work	1		1	

During start of work	1.635	0.934–2.859	1.552	0.837–2.876

None	2.246	1.442–3.496	2.104	1.267–3.493

**Provision PPE**				

Yes	1		1	

No	1.514	0.929–2.468	1.088	0.6051.956


* Adjusted for Age.

### Factor influencing occurrence of skin diseases among construction workers (multivariate regression analyses)

Prediction for skin diseases was carried out using the identified risk factors (age, education, job category, work experience, safety training on work, period of safety training, safety inspection done by site managers, provision of personal protective equipment, training and guidance on utility of PPE and frequency of wearing gloves) (***Table 3***).

After adjusting for age, carpenters showed high chances of developing skin problems (OR 11; 2.55–54.62), followed by assistant masons (OR 9.6; 2.84–32.87) and masons (OR 6.745; 2.19–20.73). Construction workers who had worked more than four years had a high chance of developing skin problems (OR 2.21; 1.28–3.82) (***Table 3***). Workers who did not receive safety training at any point were prone to skin problems (OR 2.10; 1.27–3.49). Whereas, safety inspection done by site managers, provision of PPE, training and guidance on utility of PPE and frequency of wearing gloves did not stand as influencing factors (***Table 3***).

## Discussion

This study was done to determine the prevalence of skin disease and their associated factors that influenced their occurrence and to assess the preventive measures being used by construction workers. The study found that 54% of construction workers had occupational skin disease in the preceding 12 months (***Figure 1***). Factors that were significantly associated with the occurrence of skin disease included age, duration at work and job category. Preventive measures, such as conducting safety training, the time of giving safety trainings and guidance on utility of PPE in general had an influence on the prevalence of occupational skin diseases.

### Prevalence of skin diseases

In our study, the overall prevalence of occupational skin diseases (54%) was similar to findings reported in a study done in Mangalore, where 53.74% of the construction workers reported to have skin diseases [9]. Similarly, findings among construction workers in Dhaka, Bangladesh, showed that 59.5% of construction workers reported having skin diseases [6]. Other studies done in Great Britain and Ahmedabad presented slightly lower prevalence compared to our study (17% and 47%, respectively) [5,11]. The control measure available in these two states may be the explanation for the lower prevalence. In addition, studies in Ethiopia and Bangladesh presented higher prevalence on skin diseases among female construction workers (84% and 82%, respectively) compared to 57% in our study [12,13]. A longitudinal study done in Germany had slightly high registered cases of occupational skin disease at baseline (68%) [14]. This could be due to the methodology: they used patch test to confirm the disease, while in our study we used observation; hence, some cases might have been missed.

The current study reported a prevalence of skin disease of 68% among carpenters, 64% among assistant masons and 55% among masons, with a lower prevalence among painters and plumbers (15.4%). These findings are different compared to those reported elsewhere, where 90% of masons had skin disease, followed by helpers and tile setters [15]. This difference may be attributed to the definition of skin disease between the studies and the job categories included in the study. The higher prevalence in carpenters found in our study could be due to the job task they had at that moment, such as having skin contact with various types of woods and wood preservatives, which contain several irritants and sensitizers, such as thinner, glues and turpentine, when setting doors and windows.

Various skin diseases have been reported when examining construction workers. In the study done in Kerala, India, skin burns and skin irritation were most prevalent at 55% and 52%, respectively [15], and compared to another study done in Vadodara [5], the prevalence of specific symptoms was slightly higher in our study, with such symptoms as flakes (80%), itching (78%) and fissures (70%).

In this study, construction workers aged 31 and above reported a higher prevalence of skin diseases (63%) compared to those aged 25 and below. A study done in Germany reported similar findings, which stated that age group 35 and above had a high prevalence of skin diseases (42%) when compared with the age group 34 and below [14]. Other studies reported a high prevalence of skin diseases in lower age groups: 20–25 years [5] had a high prevalence of skin disease (30%) compared to the age group 30–35 years (6%). This could be because of the minimum age group for employment between the two study locations.

The prevalence of skin diseases boomed in the group with four years or more of work experience (40%) compared to workers with less than two years and between two and three years. This was also reported in a study by Shah and Tiwari: 39% of the workers who were suffering from skin problems were involved in construction for more than six years, while a low prevalence showed on workers with five years of experience or less [5].

### Factors influencing occurrence of skin diseases

Similar to our study, studies done in Ahmedabad and Vadodara showed that work duration was a determinant for developing skin disease among construction workers [5]. Other studies also reported the same findings [3,6,16,17]. In our study, age of the participants was among factors found to influence the development of skin disease, where workers 36 years and above were more likely to develop skin disease (AOR 2.5; 1.379–4.454). This was similar to other studies [6,12,13], which reported higher prevalence among those 36 years and above. A longitudinal study done in Germany also demonstrated duration at the workplace is amongst the contributing factors leading to skin diseases, reporting that occurrence of skin diseases among construction workers became more prominent after at least 10 years of exposure [18]. Thus, both work duration and age of the workers could explain the similar findings across the world.

A carpenter was found to have higher odds of developing skin disease compared to masons and assistant masons (AOR 11; 2.60–46.78), which was different from other studies where carpenters were not included. Most studies are interested in construction workers who deal with sensitizer, such as cements [6,12,18,19]; however, our study revealed that carpenters are most prone to the development of skin diseases.

In our study, education level showed no significant influence in the occurrence of skin diseases, despite an AOR of 1.20 (95% CI 0.43–3.40) and 1.32 (95% CI 0.7–2.22) for non-formal and primary level, respectively. Similar observation was encountered in a study done in Mombasa County [3].

### Preventive measures

Our study revealed that workers who did not receive safety training at any point during work had higher chances of developing skin diseases compared to those who received training before and during the beginning of the work shift (OR 2.25; 1.44–3.50). Adisesh and co-authors reported that safety training is crucial in preventing the development of skin diseases as it helps the worker/individual to diagnose the cause of the problem and opt for solutions willingly to improve themselves in comparison to those who do not have any information. Safety training on work proved to be a crucial factor in our study: those who did not receive any safety training had an OR of 1.87 (95% CI 1.27–2.76). However, in our study it was evidenced that provision of safety training was not a key issue; what mattered was provision of training before the commencement of a task or on-the-job training. Other studies did not outline the time of providing training; they only mentioned provision of safety training and its importance [15,20,21,22,23].

Construction workers’ hands are the most exposed body part, thus frequency of wearing gloves can alter the situation. In our study, it was reported that about 52% of those who did not use PPE when performing their tasks had skin diseases. A similar study done in India reported almost 35% of construction workers suffered from skin diseases due to irregular usage of PPE [16,17]. However, it was not statistically significant for our study. This could be due to construction workers having low knowledge on the types of PPE to be used; thus, they may be given PPE but could not identify if it was the proper PPE for the job task. This can be similarly noted for glove usage.

Nevertheless, in our study, safety inspections done by management, training and guidance on utility of PPE and frequency of washing hands did not demonstrate any significant association with reducing the occurrence of skin disease, but it was reported to be amongst the preventive measures that alter the occurrence of skin diseases [24,25,26,27].

### Strengths and limitations

The current study established the prevalence of the problem, which correlated with results from other studies around the world. The Nordic Occupational Skin Questionnaire [10] used in data collection is validated, standardized and accepted for assessment worldwide, which means the results of this study are comparable with results of similar studies.

The study design was cross sectional, in which specific cause and effect are studied at the same time, and the chance of missing participants with effects were minimized by the nature of exposure. Occupational exposure can occur sometime before the effect is realized, which is also the case with occupational skin disease [18]; hence the chance of missing the outcome was minimal despite the study type. However, the use of a patch test might have revealed participants with less pronounced skin disease, and a follow-up study would have revealed true incident rates.

The study was based on self-reported symptoms and inspection by a doctor. Any chance of over- or underreporting was minimized by the doctor’s examination of the hands, the structure of the questions, as well as the confidentiality of the participants. It is believed that data collected can enhance the understanding of the whole course of development of skin disease, its associated factors and effective preventive measures.

## Conclusion

The study indicated that a high number of construction workers experience occupational skin diseases, with more seen among carpenters. However, most of the symptoms were reported in masons due to the number of participants.

A majority of the workers aged 36 years and above reported skin diseases due to their occupation, and workers who have worked for more than 4 years also reported more skin diseases than their counterparts. Safety training before commencement of works shift, inspection conducted by the management and training on PPE use, especially wearing gloves during work time, may reduce the occurrence of occupational skin diseases. The study was cross sectional; thus, a causal relationship cannot be concluded, though it can be referenced for future studies.
